# Acute Stent-Induced Endothelial Denudation: Biomechanical Predictors of Vascular Injury

**DOI:** 10.3389/fcvm.2021.733605

**Published:** 2021-10-15

**Authors:** Claire Conway, Farhad R. Nezami, Campbell Rogers, Adam Groothuis, James C. Squire, Elazer R. Edelman

**Affiliations:** ^1^Department of Anatomy and Regenerative Medicine, Royal College of Surgeons in Ireland, Dublin, Ireland; ^2^Institute for Medical Engineering and Science, Massachusetts Institute of Technology (MIT), Cambridge, MA, United States; ^3^Trinity Centre for Biomedical Engineering, Trinity College Dublin and Royal College of Surgeons in Ireland, Dublin, Ireland; ^4^Thoracic and Cardiac Surgery Division, Department of Surgery, Brigham and Women's Hospital and Harvard Medical School, Boston, MA, United States; ^5^Cardiovascular Division, Department of Medicine, Brigham and Women's Hospital and Harvard Medical School, Boston, MA, United States; ^6^HeartFlow Inc., Redwood City, CA, United States; ^7^Department of Electrical and Computer Engineering, Virginia Military Institute, Lexington City, KY, United States

**Keywords:** endothelial injury, coronary artery, endovascular stent, finite element analysis, computational fluid dynamics

## Abstract

Recent concern for local drug delivery and withdrawal of the first Food and Drug Administration-approved bioresorbable scaffold emphasizes the need to optimize the relationships between stent design and drug release with imposed arterial injury and observed pharmacodynamics. In this study, we examine the hypothesis that vascular injury is predictable from stent design and that the expanding force of stent deployment results in increased circumferential stress in the arterial tissue, which may explain acute injury poststent deployment. Using both numerical simulations and *ex vivo* experiments on three different stent designs (slotted tube, corrugated ring, and delta wing), arterial injury due to device deployment was examined. Furthermore, using numerical simulations, the consequence of changing stent strut radial thickness on arterial wall shear stress and arterial circumferential stress distributions was examined. Regions with predicted arterial circumferential stress exceeding a threshold of 49.5 kPa compared favorably with observed *ex vivo* endothelial denudation for the three considered stent designs. In addition, increasing strut thickness was predicted to result in more areas of denudation and larger areas exposed to low wall shear stress. We conclude that the acute arterial injury, observed immediately following stent expansion, is caused by high circumferential hoop stresses in the interstrut region, and denuded area profiles are dependent on unit cell geometric features. Such findings when coupled with where drugs move might explain the drug–device interactions.

## Introduction

The proliferation of drug-eluting stent designs has been accompanied by the need to understand more fully the relationship between stent design and acute arterial injury (1). Arterial geometry is an issue for all aspects of the interaction between stent, drug elution, and vascular response to injury. Understanding the relationship between stent design and acute arterial injury has become even more vital as the number of patients receiving these endovascular implants increases [>3 million globally annually (2)]. Endovascular stents have transformed the clinical outcomes for atherosclerosis treatment, and the utilization of animal models can provide significant insight into the biological response to stent implantations.

However, the precise interaction of acute vascular injury and stent geometrical features that may control this biological response is understudied. While the results of drug elution have been spectacular (3–5), there remains a concern that the impact of drugs and/or the extent of injury varies along the length of the stent. “Edge effects” leave the ends of the stent more vulnerable to residual restenosis (6). Endothelial denudation and exposure/disruption of subintimal vascular structures profoundly affect the pharmacodynamics of drug absorption and penetration (7, 8). Understanding the precise mechanical initiators of acute vascular injury may permit control of these variables for optimal drug delivery and directly guide the design and evaluation of stent geometries that minimize undesired mechanical injury (9). Several different mechanisms of injury have been reported including chronic stress from the stent struts normal to the vessel resulting in partial laceration (10), chronic changes in surface shear stress from altered blood flow that may trigger maladaptive remodeling (11, 12), and scraping of the vessel wall at the ends of the stent caused by end-first expansion (13).

None of the above mechanisms, though, explain the phenomenon of interstrut injury. First noted by Rogers et al. (14), who used the extent of retention of the endothelium as a sensitive marker of stent-induced injury, this response involves a regular pattern of endothelial denudation occurring in the center region of each repeating unit that defines the stent geometry. This injury occurs immediately poststenting and therefore cannot be a product of vascular remodeling caused by altered blood shearing stress. The pattern repeats inside each cell over the length of the stent and is thus not an artifact of end-first stent expansion. Further, denudation occurs in the center of each cell, not the perimeter, eliminating both direct strut-vessel mechanical interaction from consideration and regions of stagnant blood flow.

These findings suggest that endothelial cells are sheltered from excess strain when in proximity to the strut borders, but denude in the center where the basement membrane is more greatly distended. From a structural mechanics' perspective, device–tissue interaction is readily suited to computational investigation, which permits quantification of what is not feasible with physical experimentation.

Rogers et al. (15) examined the hypothesis that the mechanism of vascular injury during stent deployment is the balloon–artery interaction. A simplified, two-dimensional (2D) plane strain finite element analysis (FEA) indicated, for a constant interstrut distance and balloon compliance, that the effective (calculated based on 2D assumptions) surface stress reaches a maximum at the center of the cell opening primarily due to balloon–artery contact. However, advances in FEA solvers and high-performance computational power now enable this problem to be examined in three dimensions.

Advanced three-dimensional (3D) FEA techniques have enabled the prediction of the stent “dogboning” phenomenon (16, 17), stent radial recoil (16–18), axial foreshortening (18), bending stiffness (18), tissue prolapse (19), and device radial strength (18). In addition, predictions of these stent deployment metrics have been validated experimentally with optical measurements (20, 21), device radial loading (21), and device bend testing (22, 23). FEA is a powerful tool that readily accounts for geometric changes in stent design enabling optimization (24), calculates the effects of change in materials from permanent or degradable metallic alloys to more recently bioresorbable polymers (25), and with its ultimate aim, within the stenting community, to simulate successful scaffolding with minimal stress-induced vascular damage. However, the stent-artery mechanical interaction requires further investigation, which is the contention of these authors, as a 3D quantitative explanation of phenomenon of interstrut injury remains absent from the literature.

Here we reexamine some of the *in vivo* data from Rogers et al. (15) as we hypothesize that loading in the interstrut region due to stent expansion results in excessive tensile circumferential hoop stress resulting in endothelial denudation. If this hypothesis is true, we may use the stent as a testbed to determine the threshold at which denudation occurs as areas of both intact and denuded endothelium are repeatedly observed in different stent expansions. This study explains stent-induced arterial injury using *in vivo* and *in silico* techniques. It aims to explain interstrut injury in terms of a numerically derived mechanical predictor and further investigates the consequence of stent design changes on biomechanical vascular response.

## Materials and Methods

### Finite Element Analysis

**Figure 1:**
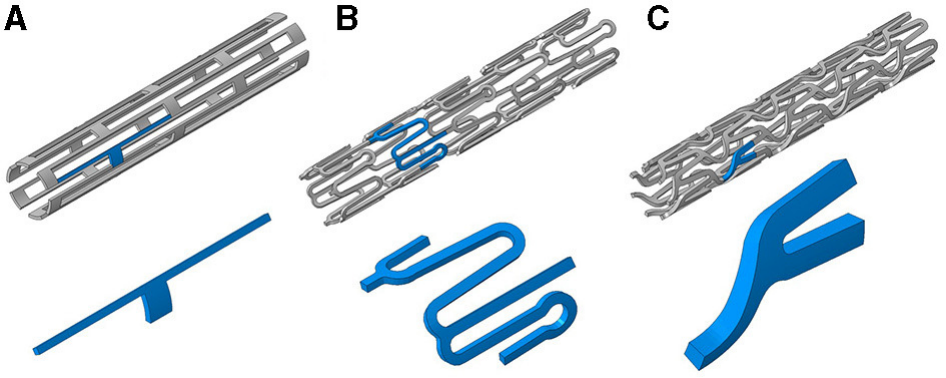
Three stent designs used in this study with inserts (blue highlighted sections) showing representative repeating units within each design: **(A)** slotted tube, **(B)** corrugated ring, and **(C)** delta wing.

The predicted arterial state due to trifolded balloon deployment of three stent designs was examined using 3D FEA techniques (Abaqus/Explicit v6.14, Dassault Systemes, RI, USA). The three studied stent geometries were the slotted tube, corrugated ring, and delta wing designs (Figure 1). Each design had a radial strut thickness based on the values reported by McClean and Eigler (26), which resulted in a 63-μm slotted tube, 56-μm corrugated ring, and 94-μm delta wing design. Each stent design was deployed via inflation of a trifolded balloon wherein, for numerical efficiency, one-half of the axial length of the assembly was modeled taking advantage of symmetry within the model setup. Each stent geometry was meshed with eight-noded, reduced integration, continuum, hexahedral elements. Mesh densities were determined by comparison with the authors' previously published sensitivity analyses for each stent design (27). The resulting meshes for the slotted tube, corrugated ring, and delta wing designs resulted in the total number of 39,168, 26,199, and 57,330 elements, respectively.

The nylon inflation balloon was similar to a design presented in the work of Mortier et al. (28). The balloon in its unwrapped configuration reached a diameter of 3 mm. The wrapping of the balloon was simulated by the method outlined by Laroche et al. (29) and explained by Conway et al. (30). The balloon geometry was meshed with four-noded, reduced integration, membrane elements. Each stent design was deployed in a straight single-layer arterial segment, which was meshed with eight-noded, reduced integration, continuum, hexahedral elements. The artery had a wall thickness of 0.5 mm and an inner diameter of 3 mm. The straight segment of artery was utilized to be comparable to the *in vivo* stent implantation conditions in the rabbit iliac artery—later detailed.

Each stent device was modeled as 316L biomedical-grade stainless steel. Stent deployment methods and stent-balloon assembly material properties have been reported previously (25, 31), and vessel tissue properties were extracted from literature (32, 33). Briefly, the elastic behavior of the stent was considered to be linear and isotropic in terms of finite deformation stress and strain measures, with a Young's modulus of *E* = 200 GPa and Poisson's ratio of υ = 0.28. Plasticity was described by isotropic hardening *J*_2_ flow theory, where the specific form of the strain hardening curve was taken from McGarry et al. (34) including a yield strength of 264 MPa and an ultimate tensile strength of 584 MPa at an engineering plastic strain of 0.247. The semicompliant nylon balloon was modeled as linear elastic with a Young's modulus of *E* = 850 MPa and a Poisson's ratio of υ = 0.4. The arterial wall was modeled as a non-linear, isotropic, hyperelastic material.

The function *W* (Equation 1) describes the second-order polynomial strain energy density function (35) implemented to describe the arterial constitutive response and is described as


(1) $$\begin{array}{l} W=\;a_{10}\left(I_{1}-3\right)+\;a_{01}\left(I_{2}-3\right)+\;a_{20}{\left(I_{1}-3\right)}^{2}\\ \;+a_{11}\left(I_{1}-3\right)\left(I_{2}-3\right) \end{array}$$

where *I*_1_ and *I*_2_ are the strain invariants, and *a*_*ij*_ are the hyperelastic constants. The hyperelastic constants were taken from the mean values reported in Lally et al. (32) with *a*_10_ = 11.438 kPa, *a*_01_ = 21.296 kPa, *a*_20_ = 601.245 kPa, and *a*_11_ = 1,205.26 kPa.

**Figure 2:**
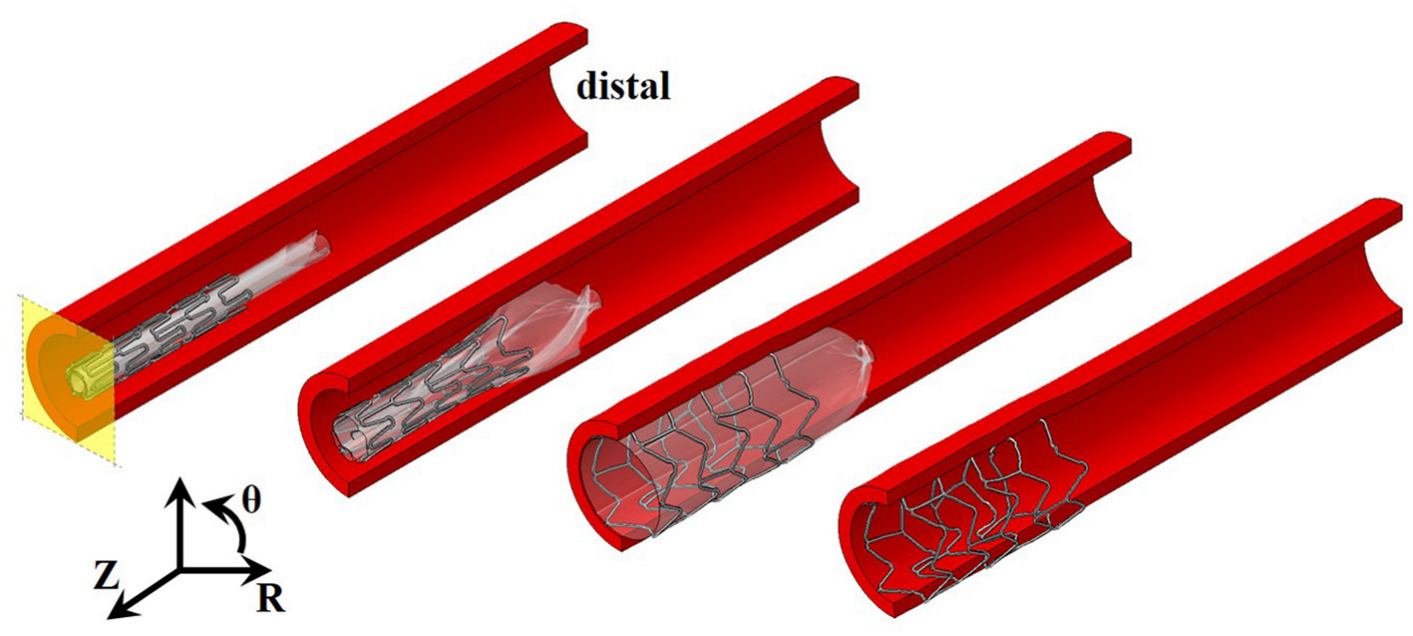
Stages of stent expansion for pressurization of a trifolded balloon. Pressure applied to the internal surface of the balloon (transparent white) results in expansion of the distal end of the stent first, and then the central region before full deployment. Axial plane of symmetry shown in yellow and cylindrical coordinate system used, where Z represents axial direction, θ represents circumferential direction, and R represents radial direction.

Mesh nodes on the stent, balloon, and arterial vessel in the axial plane of symmetry were constrained to prevent axial translation. The distal end of the balloon was pinned, and the distal end of the arterial vessel was constrained in the radial and circumferential directions (see Figure 2 for deployment set-up). A pressure of 1.8 MPa was applied to the inner surface of the folded balloon mesh for each stent expansion simulation. This inflation pressure was determined from free expansion simulations (no vessel included) for the balloon to reach its nominal diameter. A coefficient of friction of 0.2 was applied to all contacting surfaces during deployment analyses, as per previous approaches (30, 31, 36, 37).

### Experimental Validation

FEA predictions were experimentally validated by stenting both iliac arteries of six New Zealand white rabbits (3–4 kg, Millbrook Farm Breeding Laboratories, MA, USA). Experiments were conducted under a protocol approved by the Institutional Animal Care and Use Committee according to the US Animal Welfare Act and Animal Welfare Regulations code. The 12 vessels were randomly assigned to be stented with one of three designs, which comprised a slotted tube (Advanced Cardiovascular Systems/Guidant slotted tube), corrugated ring (Advanced Cardiovascular Systems/Guidant Multilink), and delta wing (Medinol Corporation, NIR). Rabbits were anesthetized with ketamine [35 mg/kg, intramuscular (IM)] and sodium pentobarbital (Nembutal, 4 mg/kg IM). Bare stainless-steel stents, mounted on 3-mm-diameter ×12-mm-length non-compliant balloon angioplasty catheters (Advanced Cardiovascular Systems/Guidant), were inserted for inflation. The balloon catheter was pressurized to a maximum inflation pressure of eight atmospheres (atm) over a 30-s time period. The arteries were harvested 5 to 15 min after stenting and stained *in situ* with silver nitrate (15, 38) or Evans blue stain (delta wing). The arteries were excised, opened longitudinally, and mounted *en face* within 4 h of harvesting. Percent of endothelial denudation was determined using computerized morphometric analysis in the annular region extending 1 mm out from the edge of the stent.

### Analysis of Strut Thickness Variation

Following comparison of the FEA deployments with *in vivo* endothelial denudation staining, a further study to investigate the effect of strut thickness on predicted biomechanical tissue response was performed. This *in silico* experiment used FEA and computational fluid dynamics (CFD) to predict the altered vascular response for variations in radial strut thickness for the slotted tube stent design. The methodology for the FEA deployments was identical to those previously described in this study, and five radial strut thicknesses were considered: 63, 75, 100, 125, and 150 μm. Following FEA deployment, the deformed meshes for the stent and vessel were used as inputs for CFD.

### Wall Shear Stress Analysis

Wall shear stress (WSS) is a universally approved metric of hemodynamic performance of endovascular implants (39, 40). Herein, we computationally investigated the effects of varying strut thickness on flow perturbations and consequent sizing of areas exposed to pathological shear stresses. As a representative case, the numerically generated deformed artery with slotted-tube stent, as elaborately explained above, was used in CFD studies. Computational grids were generated by ANSYS ICEM CFD (ANSYS, Canonsburg, PA), consisting of approximately 11 ± 0.5 million tetrahedral elements in different cases. The computational mesh is adequately refined near the stent struts and the arterial wall and coarsened toward the arterial centerline to optimize the computational costs. Independence of results from spatial discretization was checked through multiple steps of grid refinements and comparison of WSS and flow profile. Blood was simulated as an incompressible fluid, with a density of 1,060 kg/m (3), which expresses non-Newtonian behavior with shear-dependent dynamic viscosity according to the Carreau model (41, 42). Blood was assumed to enter the artery with the rate of 0.95 ml/s, similar to typical flow rate of coronaries at the ostium, and the artery outlet was set to 70 mm Hg relative pressure (43). To minimize the uncertainties introduced by boundary conditions, inlet and outlet were extended to induce fully developed flow at the former, and minimum back flow at the latter. In addition, a no-slip boundary condition was prescribed at the arterial wall and stent struts. ANSYS CFX 17. 2 (ANSYS) was used to solve the steady conservation of mass and momentum equations, which were discretized with second order accuracy. Simulations were assumed converged when the residuals reduced to 10^−6^ of the initial values. ANSYS CFD-Post 17. 2 (ANSYS) was used to extract the shear metrics and calculate the area exposed to atheroprone WSS, that is, <1 Pascal (Pa).

## Results

### Interstrut Hoop Stress Correlates With Endothelial Denudation Sites

FEA predictions were investigated as to whether high tensile hoop stress in the interstrut region correlated with sites of endothelial denudation. For each stent design deployed, the FEA predictions of arterial stress state were examined. The predictions were then compared with representative histological stains for endothelial denudation relating to the deployment of each stent design.

**Figure 3:**
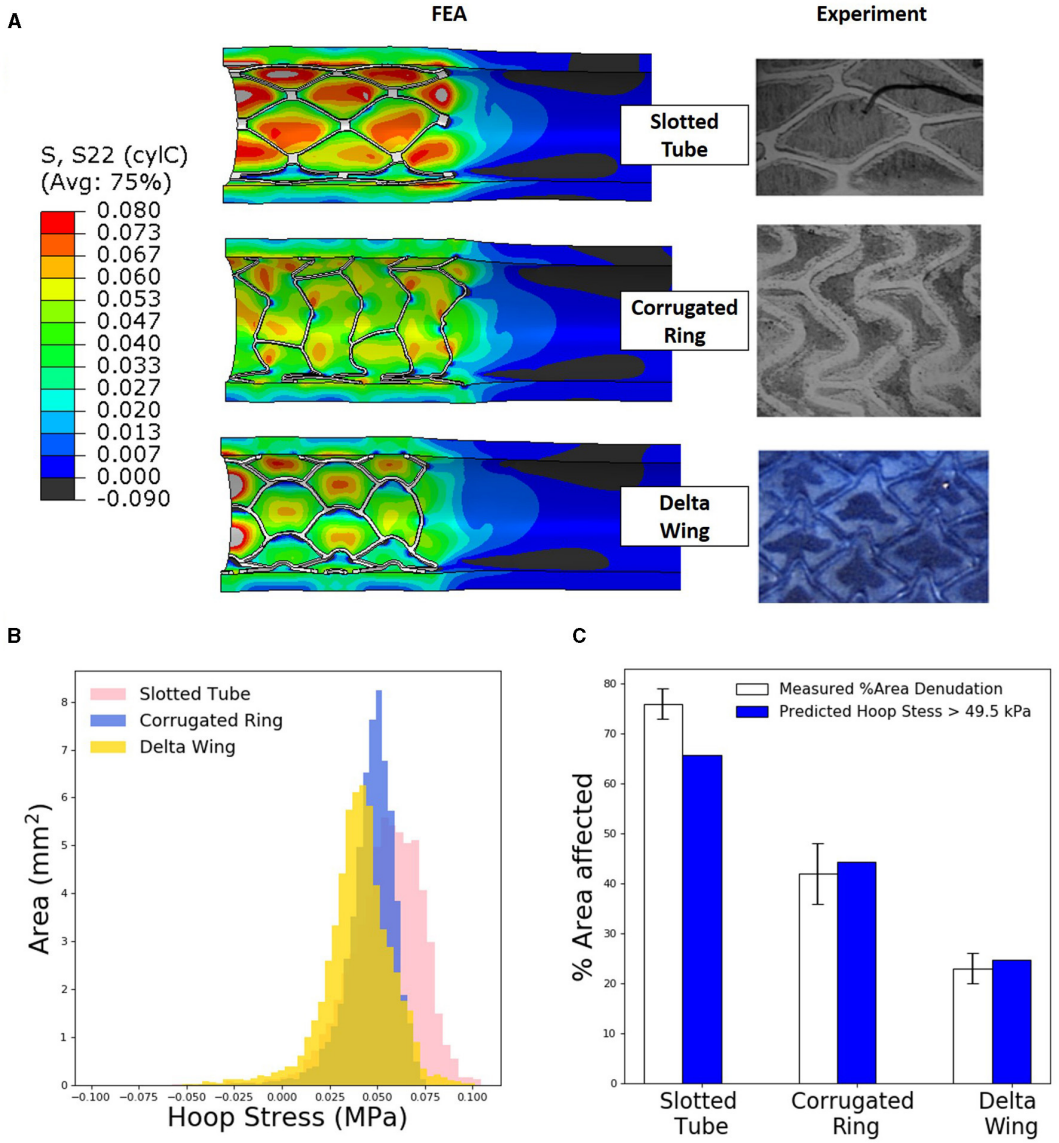
Levels of tissue hoop (circumferential) stress due to stent expansion predicts sites of endothelial denudation. **(A)** Finite element analysis (FEA) predictions of arterial hoop stress (MPa) following deployment of three different stent designs compared with representative histological stains [slotted tube and corrugated ring adapted with permission (15)] for endothelial denudation following *in vivo* deployment of each design. **(B)** Distribution of arterial luminal surface area at given levels of hoop stress for each of the three stent designs considered. **(C)** Comparison of measured percent of area denudation from computerized morphometric analysis of arterial tissue vs. FEA predicted arterial surface area exposed to a hoop stress exceeding 49.5 kPa following deployment of each stent design.

Predicted regions of high tensile hoop stress correlate with endothelial denudation following deployment of the slotted tube, corrugated ring, and delta wing stent designs (Figure 3). A threshold hoop stress was extracted in line with the hypothesis that high tensile hoop stress is predictive of endothelial denudation in the interstrut locations. Through the use of deformed nodal coordinates for a given element face, surface area and hoop stress predicted per element were extracted using custom Python postprocessing. This enabled plotting of a high-resolution hoop stress histogram to expose a threshold stress that aligned with experimental denudation measures.

Total artery luminal areas, with a hoop stress exceeding 49.5 kPa, were compared with observed endothelial denudation, which was quantified with computerized morphometric analysis for each stent design. In a slotted-tube–style stent, FEA simulations predicted a 65% area of denudation, slightly underestimating the 76 ± 3% experimentally observed. For the corrugated ring stent, FEA predicted that the area of denudation was 45% comparing well with the 42 ± 6% experimentally observed. Similarly for the delta wing stent design, FEA predicted the area of denudation to be 27% comparing well with the 23 ± 3% experimentally measured.

### Thicker Stent Struts Increase Likelihood of Endothelial Denudation

**Figure 4:**
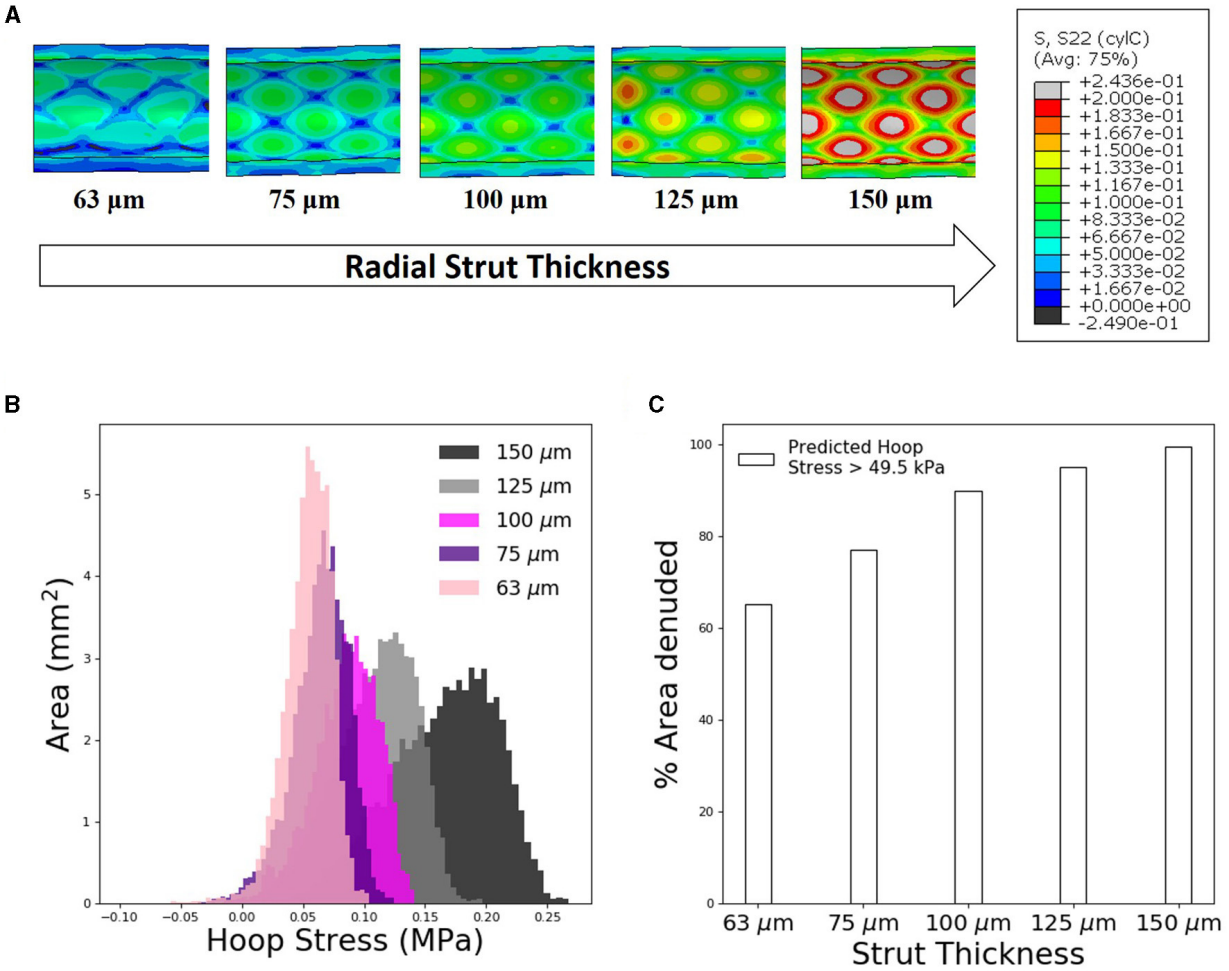
Increasing stent strut thickness increases predicted arterial interstrut hoop stress and corresponding risk of endothelial denudation. **(A)** Arterial hoop stress (MPa) contour plots following deployment of slotted tube design for a range of radial strut thicknesses: 63, 75, 100, 125, and 150 μm. **(B)** Distribution of arterial luminal surface area at given levels of hoop stress for each of the five stent strut thicknesses simulated. **(C)** Finite element analysis (FEA) predicted surface area exposed to an arterial hoop stress exceeding 49.5 kPa following deployment of each slotted tube design for varying radial strut thickness.

For the slotted-tube–style stent, devices with a range of radial strut thicknesses were deployed using FEA techniques to examine consequence of strut thickness on predicted arterial hoop stress and hence the likelihood of endothelial denudation. Stress increased with increasing the considered radial strut thicknesses (63, 75, 100, 125, and 150 μm, Figure 4). Further analysis of the hoop stress distribution, using custom Python postprocessing, showed a shift in stress distribution to higher stress states for increasing strut thickness. Using the previously calculated threshold hoop stress of 49.5 kPa to denote endothelial denudation, the predicted arterial surface area denuded increased substantially for an increasing stent strut thickness (Figure 4).

We extracted WSS as one of the most prominent factors to study the direct effect of strut thickness on flow disruption and hemodynamic changes as predictors of delayed vascular healing. Similarly, for all stented cases, we observed localized areas of atheroprone low WSS (<1 Pa) where flow separates and recirculates, that is, at the vicinity of stent struts. Regions of high WSS, on the other hand, were likely to occur on the inner side of the stent struts protruded in the lumen, facing blood with higher velocity, and thus elevated WSS. This analysis is similar to predictions of Pant and colleagues (44), where high WSS appears in the interstrut region, implications of which are discussed later.

**Figure 5:**
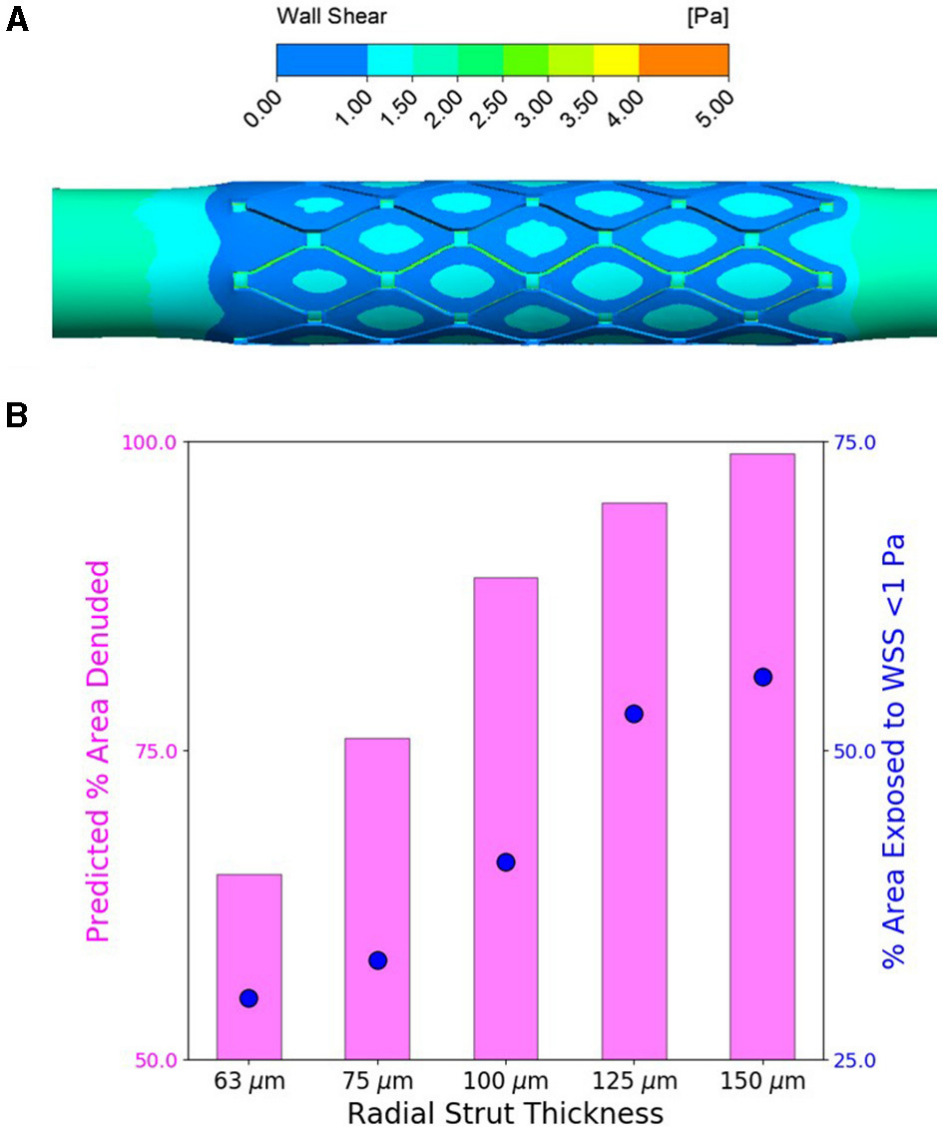
Increasing stent strut thickness increases predicted arterial luminal surface area exposed to the threshold hoop stress for endothelial denudation and also predicted area exposed to wall shear stress (WSS) <1 Pa. **(A)** Computational fluid dynamics (CFD) representative WSS contour map for flow through slotted tube (radial strut thickness of 125 μm)-stented artery. **(B)** Percent of arterial luminal area exposed to WSS <1 Pa (shown in blue) for increasing strut thickness compared to percent of area likely to be denuded from finite element analysis (FEA) predictions (shown in pink) for increasing strut thickness.

We also observed for all cases, independent of the stent thickness, larger areas exposed to low WSS at proximal edges of stents, as those areas were geometrically configured as a sudden expansion due to stenting, thus introducing flow separation and recirculation (Figure 5A). More importantly comparing the areas exposed to atheroprone low WSS, we clearly noticed a direct relation to strut thickness, wherein stents with thicker struts induced bigger areas of low WSS at stented regions.

## Discussion

Drug-eluting stents continue to evolve with the persistence of cardiovascular disease. The stents shore the artery open, preventing collapse, and the drug mediates the natural response to localized stress. Yet, there are still instances when the biology goes awry, and continued innovation emerges. Though for the first time in a while potential novel ideas have not advanced the clinical armamentarium. Local delivery has been called into question with lingering, though now receding, concern that paclitaxel in particular when administered from stents or balloons can increase mortality, and attempts at major revision of materials have failed. The attraction of a bioresorbable vascular scaffold received Food and Drug Administration (FDA) approval, but subpar clinical performance led to device withdrawal (45) as these polymeric scaffolds had a significantly higher rate of definite/probable thrombosis at 30 days after implant compared to permanent metallic DES (46, 47). But work in the area of absorbable polymeric and metallic vascular scaffolds continues, with a renewed focus on devices with sub−100-μm struts (48). It is likely that improved clinical outcomes can be achieved with these devices in the future.

It is clear that there is still much to be learned even with all we have learned about endovascular implants. The withdrawn bioresorbable scaffolds were 150-μm-thick, and we do know that scaffold thrombosis is linked to strut thickness through reduction in WSS and increased flow separation and flow stagnation (49). Such flow disruptions enhance platelet deposition and thrombin and fibrin generation in the strut vicinity, and numerically, a hemodynamic marker of this is low WSS (50). Yet what remains unknown is how immediate vessel injury poststenting, characterized by endothelial denudation, is linked to stent design. This work highlights the links between device design and biological consequence in terms of biomechanical tissue stress state as a marker of endothelial denudation. In addition, the predicted consequence of strut thickness changes on the WSS (as a marker of delayed healing) and tissue circumferential stress (as a marker of denudation) were examined.

In fact, our work lies within the spirit of stent design guided by physiologic mechanistic insight under a computational lens. The desire to maximize the postintervention arterial lumen area with the smallest endovascular implant footprint has been accompanied by understanding that this is not accomplished by overinflation alone. Stent design well-beyond stent dimensions is an important determinant of reactive intimal hyperplasia and thrombosis (38, 49), balancing material properties such as fatigue and recoil with vascular repair. Advances in vascular biology and computational methods have enabled our community to consider an ever-expanding spectrum of complex device designs, reliably predict *in vivo* effects, and quantify the link between mechanical metrics and *in vivo* observations for validation. So too it is here—interstrut stress can now be added to the array of design-imposed forces on the vessel wall, adding to the array of factors we have already defined as essential in stent biology.

FEA allows prediction of vessel mechanical stresses as a result of stent implantation. High mechanical stresses are hypothesized to be predictive of vascular injury sites, which may act to induce a cascade of biological events leading ultimately to in-stent restenosis. Typically, studies using FEA to predict stented-artery stress state focus on von Mises stress as an output variable (17, 51–53), but this positive scalar does not account for directionality. Others have shown contour plots of radial, circumferential, and axial components of arterial stress in their FEA assessments of expanding stents (19, 54). Yet, it remains to be precisely predicted and validated with quantitative biological evidence of tissue damage. In our study, we now compare the circumferential arterial stress predictions directly with biological evidence of vascular injury in the form of denuded endothelium.

FEA predictions of arterial biomechanical state compare well with patterns observed in histological staining of endothelial denudation, from previous (15) data sets supplemented with the delta wing design. Indeed, regions of high arterial hoop (circumferential) stress correlate with regions of endothelial denudation in the interstrut regions of each of the three stent designs we considered (Figure 3A). Most pronounced in the slotted tube design, where the highest hoop stresses evident in the interstrut region, followed by the delta wing showing higher hoop stress in the interstrut region, and lastly the corrugated ring hoop stresses were predicted to show lower amounts of high hoop stresses. The complexity of the corrugated ring stent design resulted in a less regular pattern compared with the two other designs, both experimentally and numerically. Upon close examination of the predicted hoop stress distribution for each stented arterial segment, hoop stresses exceeding a threshold of 49.5 kPa in the interstrut region appeared to be predictive of endothelial denudation when compared with measured percent area denudation from both qualitative and quantitative histological analyses (Figure 3C). For the first time, we validated biomechanical hoop stress as a marker of arterial injury (in the form of endothelial denudation) immediately poststenting.

Using CFD, we and others (44) have shown predicted regions of elevated WSS appear in the interstrut region. However, this does not explain the temporal phenomenon of endothelial denudation as our previous work has illustrated (14). Briefly, endothelial restoration occurred within stented rabbit iliac arteries between 1 h and 3 days after stenting, while the hemodynamic pattern of elevated WSS predicted would stay repeatable, thus implying that damage would reoccur. Although these elevated shear rates are critical to endothelial healing and repair, shear alone cannot explain endothelial damage on implantation. The forces we are referring to are in addition to and not in lieu of the other forces that are part of the complexity of vascular repair after endovascular intervention. Within this work, our temporal force of interest was the structural component of stress to explain damage within the endothelium immediately poststenting.

Interestingly, each device was deployed to the same diameter, numerically and experimentally, and stent cell design dominated over strut thickness. The delta wing design with the thickest struts (94 μm) resulted in the least amount of endothelial denudation experimentally and the lowest levels of threshold-exceeding hoop stress numerically. This demonstrates the critical role stent unit cell geometry plays in altering the biomechanical vascular response.

Furthermore, we used computational investigation to examine the consequence of strut thickness changes on the biomechanical arterial state, following deployment of the slotted tube design. Using FEA, increasing the radial strut thickness resulted in predictions of increased circumferential stress in the interstrut region for the slotted tube design (Figure 4A). Using the previously determined threshold hoop stress of 49.5 kPa, this indicated an increase in percent of area denuded for increased stent strut thickness (Figure 4C). This would suggest that thicker stent struts result in increased arterial hoop stress, increasing endothelial denudation in the interstrut region, resulting in increased arterial injury immediately poststenting. Also examined was the consequence of these strut thickness changes on the WSS distribution using CFD, as a complementary analysis. Considering detrimental low WSS as <1 Pa, it was predicted that the percent of arterial areas exposed to low WSS increased with increasing stent strut thickness, for the slotted tube design (Figure 5B), suggesting that thicker stent struts result in more arterial tissue exposed to thrombogenic conditions.

The choice of a single-layer arterial vessel numerically is a simplification, given that arterial vessels are trilayer. Because of lack of layer-specific properties reported for animal tissue, we used the available animal mechanical data due to the nature of the *in vivo* model. Also, it should be acknowledged that further balloon calibration could be performed to match experimental and numerical balloon pressures. However, balloon diameter significantly affects the tissue stress [see Ragkousis et al. (55)], and thus, numerical attempts similar to ours attempt to replicate the balloon diameter rather than the pressure. Accordingly, as the study goal was to replicate experimental and computational nominal balloon diameters, achieving this with different experimental and numerical pressure values was deemed acceptable for the purposes of this study. This study has shown that excessive circumferential stress is predictive of denudation in the rabbit iliac. Different animal vessels would necessitate similar evaluation for species-specific threshold calculation; however, as a design tool, this study shows that excessive tensile circumferential stress could prove a valuable assessment variable for new and emerging endovascular stent designs.

However, this quantitative analysis, once again, underlines the tradeoff between structural potency of a stent and its radial scaffolding power induced by the bulk of the stent strut; this is in contrast to the hemodynamic suitability of the device. In other words, although the more recent philosophy of stent design is aligned with a low profile concept (56), more emergent bioresorbable devices have a lesser ability to scaffold highly calcified lesions and therefore entail thicker struts, which inherently delay vascular healing. Here we use FEA in combination with biological evidence to validate for the first time stented-vessel stress state. This approach has the potential to be used as a design feature for coronary stents to inform existing and future cardiovascular implant designs.

Clinically, atherosclerotic lesions are highly heterogeneous, both in terms of constituents and morphology. As our *in vivo* model was without disease, our numerical model was also without lesions. However, it would be expected to observe endothelial denudation in the interstrut region should a physical lesion be present. Using vascular hoop stress as a comparator for stent assessment strategies in atherosclerotic vascular models could assist in delineation of the suitability of a particular design for a given lesion.

In summary, this study highlights that fundamental engineering design factors have a significant effect on the biomechanical response *in vivo* almost immediately upon implantation. These effects add to the appreciation as to how mechanical forces such as flow, shear, and impaction from support materials on underlying tissues induce injury and affect healing over time. In particular, excessive interstrut circumferential stress appears predictive of the acute arterial injury that is manifest as arterial endothelial denudation. Moreover, it was shown that increasing strut thickness, in addition to delaying the healing as a result of enlarging the tissue area exposed to the atheroprone shear stresses, increases interstrut circumferential stress and thus the likelihood of endothelial denudation, bringing together the rich literature on stent design and flow/mechanical effects of expansion and retained implantation on arterial injury and repair.

## Data Availability Statement

The raw data supporting the conclusions of this article will be made available by the authors, without undue reservation.

## Ethics Statement

The animal study was reviewed and approved by MIT Committee on Animal Care.

## Author Contributions

JS and EE: conceptualization. CR, AG, JS, FN, and CC: methodology and investigation. JS, FN, and CC: writing- original draft preparation. EE, FN, and CC: writing—review and editing. All authors contributed to the article and approved the submitted version.

## Conflict of Interest

CR is employed by the company HeartFlow Inc. The remaining authors declare that the research was conducted in the absence of any commercial or financial relationships that could be construed as a potential conflict of interest.

## Publisher's Note

All claims expressed in this article are solely those of the authors and do not necessarily represent those of their affiliated organizations, or those of the publisher, the editors and the reviewers. Any product that may be evaluated in this article, or claim that may be made by its manufacturer, is not guaranteed or endorsed by the publisher.
